# Factors Affecting Health-Related Quality of Life among Survivors of Non-Hodgkin Lymphoma: A Population-Based Study

**DOI:** 10.3390/cancers15153885

**Published:** 2023-07-30

**Authors:** Stephane Kroudia Wasse, Morgane Mounier, Emerline Assogba, Cédric Rossi, Johan Adnet, Sophie Gauthier, Stephanie Girard, Kueshivi Midodji Atsou, Tienhan Sandrine Dabakuyo-Yonli, Marc Maynadie

**Affiliations:** 1 Registry of Hematological Malignancies of Côte d’Or, Dijon-Bourgogne University Hospital, F-21000 Dijon, France; morgane.mounier@u-bourgogne.fr (M.M.); sophie.gauthier@u-bourgogne.fr (S.G.); stephanie.boulanger@u-bourgogne.fr (S.G.); kueshivi-midodji.atsou@u-bourgogne.fr (K.M.A.);; 2 INSERM, UMR1231, Bourgogne Franche-Comté University, F-21000 Dijon, France; eassogba@cgfl.fr; 3 Breast and Gynaecologic Cancer Registry of Côte d’Or, Georges François Leclerc Comprehensive Cancer Centre, F-21000 Dijon, France; 4 Clinical Hematology Unit, Dijon Bourgogne University Hospital, F-21000 Dijon, France; cedric.rossi@chu-dijon.fr; 5 Methodology Biostatistics and Data-Management Unit, Georges François Leclerc Comprehensive Cancer Centre, F-21000 Dijon, France; jadnet@cgfl.fr; 6 National Quality of Life and Cancer Clinical Research Platform, F-21000 Dijon, France

**Keywords:** patient-reported outcomes, survivorship, quality of life, population-based data, Non-Hodgkin Lymphoma, diffuse large B cell lymphoma, follicular lymphoma

## Abstract

**Simple Summary:**

Survival rates among Non-Hodgkin Lymphoma patients have significantly improved in recent years. However, the impact of Non-Hodgkin Lymphoma persists in survivors. The aim of this population-based study was to describe Health-Related Quality of Life and to identify the association between sociodemographic, clinical and psychosocial factors, and self-reported Health-Related Quality of Life among Non-Hodgkin Lymphoma survivors. In total, 251 patients received the questionnaires, of whom 157 responded (63%). The main factors found to be associated with poor HRQoL of NHL survivors were age, sex, presence of anxiety, depression and economic problems. These findings suggest the need for supportive care to improve Health-Related Quality of Life and the consideration of these problems when developing care plans for Non-Hodgkin Lymphoma survivors.

**Abstract:**

Purpose: To describe Health-Related Quality of Life (HRQoL) and to identify the association between sociodemographic, clinical and psychosocial factors, and self-reported HRQoL among NHL survivors. Methods: The data of the cancer registry specialized in hematological malignancies in Côte d’Or (France) were used to identify all patients diagnosed with follicular lymphoma (FL) and diffuse large B-cell lymphoma (DLBCL) from 2010 to 2017. Patients were invited to complete SF-12 and other questionnaires. Results: The HRQoL of NHL survivors was poorer than that of the French general population (*p* < 0.05) in vitality (48 vs. 56), general health (56 vs. 63), role physical scores (60 vs. 70), role emotional scores (64 vs. 72) and the Mental Component Scale (45 vs. 49). The mean difference in physical functioning decreased per unit increase in age (β = −1.1 (0.3); *p* < 0.001). Men had better vitality than women (β = 12.4 (6.1); *p =* 0.04) and the high education level was associated with greater role emotional scores (β = 14.1 (5.4); *p =* 0.01). Symptoms of anxiety and depression were associated with poorer HRQoL. The satisfaction of social support was associated with significantly greater scores on mental health (β = 17.3 (5.1); *p* = 0.001) and social functioning (β = 15.7 (7.8); *p =* 0.04). Socioeconomic deprivation was associated with poorer general health (β = −12.8 (5.2); *p =* 0.01). Conclusions: From 3 to 11 years post-diagnosis, the main factors found to be associated with poor HRQoL of NHL survivors were age, sex, presence of anxiety, depression and economic problems. These findings suggest the need for supportive care to improve HRQOL and the consideration of these problems when developing care plans for NHL survivors.

## 1. Introduction

Non-Hodgkin Lymphoma (NHL) is the largest group of hematological malignancies (63%) in France. Aggressive NHL such as diffuse large B-cell lymphoma (DLBCL) and indolent NHL such as follicular lymphoma (FL) are common subtypes of NHL—approximately 18% and 11%, respectively [1]. Survival rates of NHL patients have improved due to advances in treatment, with the introduction of the anti-CD20 monoclonal antibody (rituximab), added to the CHOP chemotherapy regimen (cyclophosphamide, doxorubicin, vincristine, and prednisone) [2,3,4]. The 5-year relative survival in Europe was reported to be 72% for FL and 51% for DLBCL (2000–2007), with a 5-year relative survival of 86% for FL and 61% for DLBCL in France (1989–2018) [5,6,7]. However, late physical effects due to the toxicity of treatment, including cardiac dysfunction, neuropathy and metabolic complications, have been identified [8,9]. Moreover, issues such as psychosocial well-being, socioeconomic status and sexual problems tend to persist in the long term and may impair the quality of life of NHL survivors [10,11,12,13,14,15].

In this context, the question of health-related quality of life (HRQoL) in NHL patients is garnering increasing interest. HRQoL is a multi-dimensional concept that encompasses subjective perceptions and symptoms of patients as assessed by psychometric instruments [16,17]. Some studies have investigated the HRQoL of NHL survivors. Smith et al. compared non-active NHL patients with NHL patients on active treatment, to assess HRQoL in a US population-based study using different tools including the 36-item Short Form health survey (SF-36). It was shown that survivors with active disease had worse physical and mental well-being due to a more negative impact of their NHL. However, in this study, the authors did not use the eight dimensions of the SF-36 to investigate HRQOL, but rather the summary scales [18]. In France, Ben Diane et al., using the 12-item Short Form health survey (SF-12), reported that five years after a cancer diagnosis, NHL patients had more impaired physical and mental HRQoL than the general population. However, the study was based on health insurance data, which are less representative than population-based data [19].

To the best of our knowledge, few data on NHL survivors are available from population-based surveys in France. To improve our knowledge of NHL survivors in real life, we conducted a population-based study of HRQoL among survivors of NHL using a French population-based cancer registry specialized in hematological malignancies. The aim of our study was to describe HRQoL and to assess the association between sociodemographic, clinical and psychosocial factors, and self-reported HRQoL among NHL survivors.

## 2. Materials and Methods

### 2.1. Study Participants

A population-based, cross-sectional survey was conducted at the Cancer Registry specialized in hematological malignancies in the Côte d’Or area (a French Department with a total of 532,901 residents in 2019). Patients diagnosed with DLBCL or FL between 2010 and 2017, who were still alive on 1 March 2021, with an updated address, were identified. The diagnosis of DLBCL and FL was defined according to the third edition of the International Classification of Diseases for Oncology (ICD-O-3) [20].

Vital status (dead or alive) was updated using the medical files and the administrative data on death certificates to complete missing data. Death status was updated in March 2021. Other forms of hematological malignancies at diagnosis and subjects aged under 18 years were not eligible for this study. Adults with a wrong postal address, unable to express their consent and those who refused to participate were not included in this study.

This study was performed in accordance with the declaration of Helsinki. It was approved by the French national data protection authority (CNIL-MR003 N°2210227-V0) and by the Ethics committee (CPP) South-East III under the number 2020-A03479-30.

### 2.2. Data Collection

In March 2021, a letter containing the study information leaflet, the study questionnaires and a prepaid return envelope was sent to all patients identified as eligible. In the letter, it was explained that, by returning a completed questionnaire, the patient agreed to participate. Patients were reassured that non-participation did not have any consequence for their follow-up care. For patients who did not respond within one month, a reminder letter was sent together with an additional copy of the questionnaire. The questionnaire responses were anonymous, and coded a random number on each questionnaire to link to the Cancer Registry database.

### 2.3. Measurements

The Cancer Registry specialized in hematological malignancies in the Côte d’Or area routinely collects data on patient characteristics (gender, date of birth), tumor characteristics (histology, date of diagnosis, Ann arbor stage), treatment and relapse after treatment. The main outcome of this study was HRQoL, as assessed by the French-language version of the 12-item Short Form health survey (SF-12). The SF-12 is a generic questionnaire which generates eight scales, namely: physical functioning, role physical, bodily pain, role emotional, vitality, social functioning, mental health and general health, and two summary scores, the physical component summary (PCS) and the mental component summary (MCS). All scales were scored according to the standard method of the SF-12 scoring manual. Each score ranges from 0 to 100, with higher scores representing a better level of HRQoL. A score of 50 or less on the PCS has been recommended as a cut-off to determine a physical condition; while a score of 42 or less on the MCS may be indicative of clinical depression. The test–retest reliability of the PCS summary measures was 0.890 in the US and 0.864 in the UK, Coefficients of 0.760 in US and 0.774 in UK were observed for the MCS [21,22]. Psychosocial and economics factors were assessed by validated instruments, namely the Hospital Anxiety and Depression Scale (HADS), the six-item Sarason social support (SSQ6) questionnaire, and the Assessment of Precariousness and Health Inequalities for Health Examination Centers (EPICES) score.

The HADS questionnaire, validated and adapted in French in 1989 by Lepine et al., was used to determine the presence of anxiety and depressive disorders. This scale has 14 items, 7 for anxiety and 7 for depression, all rated from 0 to 3. Total scores range from 0 to 21. A score greater than 11 indicates the presence of anxiety or depression [23].

The SSQ6 questionnaire, validated and adapted in French by Rascle et al. in 2005, measures the availability of social support and the individual’s satisfaction with the perceived support. Availability scores range from 0 to 54, and satisfaction scores range from 6 to 36. A higher satisfaction score represents better perceived social support [24].

The EPICES score (“Assessment of Precariousness and Health Inequalities for Health Examination Centers”) was used to determine the level of social deprivation. This questionnaire, validated in France, comprises 11 items, each with 2 possible answers (yes/no), generating an individual deprivation score. The score varies from 0 to 100. A score > 30 constitutes a high level of social and/or material deprivation [25].

The study questionnaires also included questions on marital status, educational level, weight and height and current comorbidity. Sexuality was described on the basis of the questionnaire used in the French national study [19].

### 2.4. Statistical Analysis

To assess potential selection bias, we compared respondents and non-respondents from routinely collected data of the Cancer Registry specialized in hematological malignancies in the Côte d’Or area. Data from respondents are described using mean (SD) and median (IQR) for quantitative variables, and number (percentage) for categorical variables. We used Fisher’s exact test for categorical variables and the Mann–Whitney test for continuous variables. A *p*-value < 0.05 was considered statistically significant.

HRQoL, anxiety and depression, social support and deprivation scores were calculated, and described. We also recorded BMI into 4 categories according to the WHO recommendations [26], SSQ6 score (categorized as <median and ≥median), and lymphoma Ann Arbor stage (categorized as stage I–II and stage III–IV) [27]. HRQoL is described in the overall population, and separately in the FL and DLBCL groups. The Mann–Whitney test was used to compare continuous variables and the chi-square test was used to compare categorical variables between FL and DLBCL.A multivariate linear regression model was built to identify independent associations between sociodemographic (age, gender), clinical (time since diagnosis, Ann Arbor stage, comorbidity, BMI) and psychosocial factors (anxiety, depression, social support, economic deprivation) and each scale of the SF-12 questionnaire. Independent variables were selected based on a priori knowledge on risk factors of HRQoL [28]. The backward elimination method with a *p*-value criterion of 0.157 was used to select the predictors to be included in the multivariable models [29]. Correlations were tested between candidate covariates, at a significance level fixed at 0.05. Additionally, a complementary analysis after multiple imputation was performed to account for missing data. We imputed missing data 20 times to produce 20 complete datasets. We chose variables of theoretical interest: age, sex, time since diagnosis and histology type as predictors. A linear regression model was fitted to these variables. Proc mi- analyze was used to combine the results. The significance level for the multivariable analysis was fixed at *p*-value < 0.05 for each scale of the SF-12. All analyses were performed using SAS version 9.4 (SAS Institute Inc., Cary, NC, USA).

## 3. Results

### 3.1. Characteristics of the Study Population 

**Figure 1:**
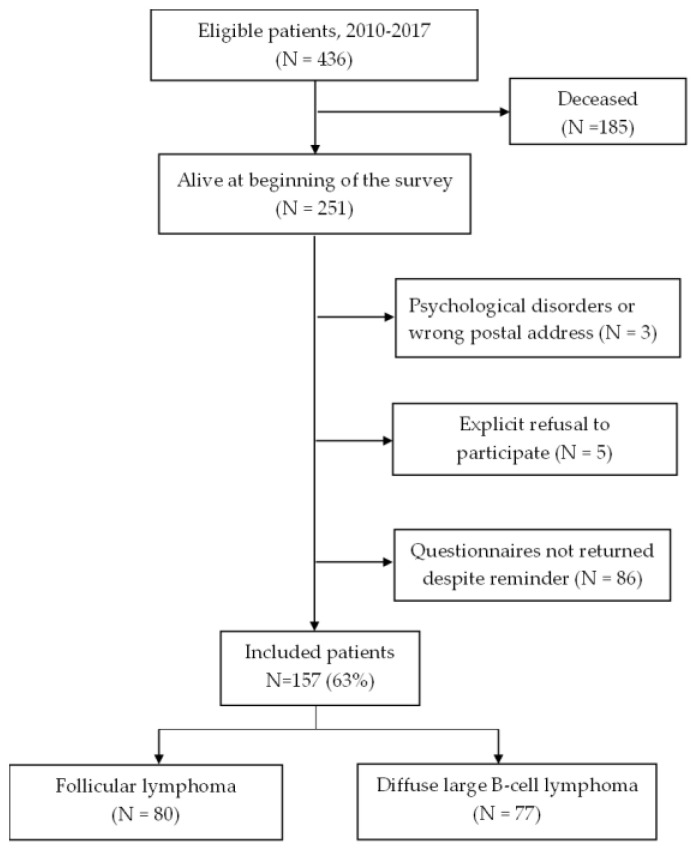
Flow chart of the study population.

Among 436 patients diagnosed with FL and DLBCL, questionnaires were sent to 251 survivors, of whom 157 completed the questionnaires, yielding a response rate of 63% (Figure 1). There was no significant difference in age between respondents and non-respondents. Non-respondents were more often women (62% vs. 45%; *p* = 0.01) and DLBCL survivors (62% vs. 49%) than respondents (Table 1).

Table 2 shows that most respondents were married or living maritally (73%) and 48% had a university level of education. The median time since diagnosis was 6 years [4,5,6,7,8], more than half were at Ann Arbor stage III–IV (61%) and did not have a relapse after treatment (92%). Overall, 32% of patients had economic problems, 46% had decreased sexual desire, 14% had symptoms of anxiety and 6% had depressive symptoms as assessed by the HADS. There was no difference between FL and DLBCL survivors, except for treatment by R-CHOP chemotherapy (57% vs. 99%; *p* < 0.0001).

**Figure 2:**
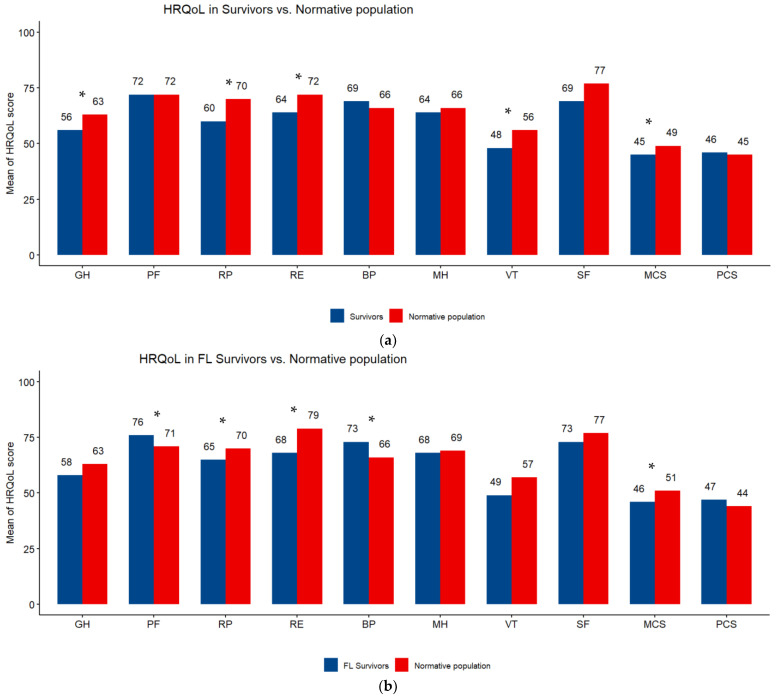

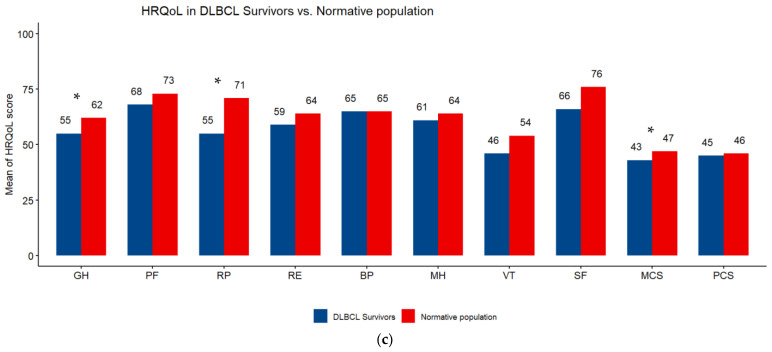
(**a**) Subscale scores on the SF-12 questionnaire. Differences between survivors of Non-Hodgkin Lymphoma and a sex- and age-matched normative population. HRQoL: health-related quality of life; PF: physical functioning; RP: role limitations/physical health; RE role limitations/emotional problems; VT: vitality; MH: Mental Health; SF: social functioning; BP: bodily pain; GH: general health; MCS: Mental Component Scale; PCS: Physical Component Scale. Higher scores are indicative of better HRQoL. * *p* < 0.05. (**b**) Subscale scores on the SF-12 questionnaire. Differences between survivors of follicular lymphoma (FL) and a sex- and age-matched normative population. HRQoL: health-related quality of life; PF: physical functioning; RP: role limitations/physical health; RE role limitations/emotional problems; VT: vitality; MH: Mental Health; SF: social functioning; BP: bodily pain; GH: general health; MCS: Mental Component Scale; PCS: Physical Component Scale. Higher scores are indicative of better HRQoL. * *p* < 0.05. (**c**) Subscale scores on the SF-12 questionnaire. Differences between survivors of diffuse large B-cell lymphoma (DLBCL) and a sex- and age-matched normative population. HRQoL: health-related quality of life; PF: physical functioning; RP: role limitations/physical health; RE role limitations/emotional problems; VT: vitality; MH: Mental Health; SF: social functioning; BP: bodily pain; GH: general health; MCS: Mental Component Scale; PCS: Physical Component Scale. Higher scores are indicative of better HRQoL. * *p* < 0.05.

Overall, the highest mean HRQoL score was on the physical functioning scale (72) and the lowest was on the vitality scale (47.6). All subscale scores were higher in FL than DLBCL survivors (Table 3). The comparison of SF-12 scales of NHL survivors with those of the normative sample from the French general population showed a significant difference (*p* < 0.05) in favor of the general population in vitality (48 vs. 56), general health (56 vs. 63), role physical (60 vs. 70), role emotional (64 vs. 72) and MCS (45 vs. 49). FL survivors had better HRQoL than the general population, the difference was significant (*p* < 0.05) for bodily pain (73 vs. 66) and physical functioning (76 vs. 71). DLBCL survivors had poorer HRQoL than the general population, the difference was significant (*p* < 0.05) for general health (55 vs. 62), role physical (55 vs. 71) and MCS (43 vs. 47) (Figure 2a–c).

### 3.2. Factors Associated with the Health-Related Quality of Life

The results of the multivariable analysis reporting the factors significantly associated with HRQoL are presented in Table 4. Age was associated with poorer physical functioning (β = −1.1 (0.3); *p* < 0.001), PCS (β = −0.3 (0.08); *p =* 0.002) and greater MCS (β = 0.2 (0.09); *p =* 0.01). Men had better vitality than women (β = 12.4 (6.1); *p =* 0.04) and the high education level was associated with greater role emotional scores (β = 14.1 (5.4); *p =* 0.01). Patients with symptoms of anxiety were more likely to have poor general health (β = −14.3 (6.3); *p* = 0.02), mental health (β = −27.1 (6.1); *p* < 0.0001) and MCS scales (β = −12.1 (3.4); *p =* 0.001). Presence of depressive symptoms was associated with significantly poorer scores on the vitality (β = −36.6 (11.1); *p =* 0.002), social functioning (β = −32.1 (13.8); *p =* 0.02) and MCS scales (β = −16.3 (4.9); *p* = 0.001). The satisfaction of social support was associated with significantly greater scores on mental health (β = 17.3 (5.1); *p* = 0.001) and social functioning (β = 15.7 (7.8); *p =* 0.04). Socioeconomic deprivation was associated with poorer general health (β = −12.8 (5.2); *p =* 0.01).

A complementary multivariable analysis was carried out after multiple imputation on missing data. The results were almost identical to the main analysis (Appendix A Table A1).

## 4. Discussion

This population-based study provides a snapshot of HRQoL and the association between sociodemographic, psychosocial and clinical factors and HRQoL in NHL survivors at 3 to 11 years post-diagnosis.

Our study found that NHL survivors had a poorer physical condition and did not have clinical depression. In comparison to the French general population, NHL survivors had poorer general health, vitality and mental HRQoL. Similar findings have been reported in France. Ben Diane et al. reported that five years after a cancer diagnosis, NHL patients had more impaired physical and mental HRQoL than the general population [19]. In the Netherlands, Mols et al. showed that from 5 to 15 years after diagnosis, the general health perceptions and vitality levels of NHL survivors remained significantly lower than those of their peers in the general population [30]. Our study also provides further insights into the HRQoL of FL and DLBCL survivors. We had hypothesized that patients with DLBCL, which is an aggressive form of NHL, would have poorer HRQoL compared to patients FL, which is an indolent form of NHL. The indolent progression of FL may not require aggressive treatment by chemotherapy and immunotherapy, depending on the case. Indeed, the “watch-and-wait” approach established by the Stanford group in the early 1980s is a conservative approach to the treatment of a select group of patients with indolent NHL [31]. In contrast, those with aggressive NHL such as DLBCL require more aggressive treatment. Our results partially support our hypothesis. Indeed, we found that all subscale HRQoL scores were higher in FL than DLBCL survivors. This may be explained by the fact that 99% of our patients with DLBCL had required chemotherapy and immunotherapy compared to 57% of FL patients.

Several factors may explain the poor HRQoL observed such a long time after diagnosis, including the impact of sociodemographic, clinical and psycho-social factors. Indeed, Immanuel et al. found that age was significantly negatively correlated with global QoL, physical and role functioning [32]. Furthermore, time since diagnosis was associated with quality of life after cancer. Wang et al. reported that compared with individuals without cancer, cancer survivors in remission of some types of cancer, such as breast cancer, colorectal cancer and melanoma, may have a similar level of HRQOL after 10 years, while survivors of prostate or cervical cancer still had lower levels of HRQOL. Survivors of hematologic malignancies had a significantly lower physical health between 2 and 9 years. [33]. Chronic conditions may be associated with increased healthcare use, medical expenditure, and lost productivity in cancer survivors. Guy et al. demonstrated that survivors of cancer are more likely than individuals without a history of cancer to have other chronic conditions, with 12.7% reporting four or more chronic conditions in addition to cancer [34]. Moreover, Vissers et al. found that among NHL survivors, comorbidities were significantly associated with lower physical and emotional function, all estimates were in the same direction whereby more comorbidities resulted in lower physical and emotional function [35]. Pettengell et al. showed that patients with relapsed disease had the lowest scores on several HRQoL dimensions. Furthermore, they compared patients on and off chemotherapy, and found that participants receiving chemotherapy reported worse scores on the overall HRQoL scale [36]. Psychosocial effects must be considered when exploring the factors associated with HRQoL, Indeed Lekdamrongkul et al. showed that when NHL survivors had lower anxiety and depression, the HRQoL score was higher [37]. Moreover, low incomes may be associated with poor HRQoL. This view was supported by a cross-sectional study that identified predictors of HRQoL in NHL survivors, finding that financial difficulties related to the disease and its treatment were clinically meaningful problems for NHL survivors and were strongly predictive of deteriorated functioning and global HRQoL [38].

Our multivariable regression analyses revealed that among NHL survivors, sociodemographic and psychosocial factors were significantly associated with HRQoL. The mean difference in physical functioning decreased per unit increase in age. This may be related to the high age of NHL survivors, as the median age in our study was 69 years. Men had better vitality than women. Our findings suggest that physicians should be aware of possible sex difference in NHL survivors. There may be a need for support services that focus on women’s needs. Patients with symptoms of anxiety were more likely to have poor general health and mental health. The presence of depressive symptoms was associated with significantly poorer scores on the vitality, social functioning and MCS scales. Similar findings have been reported, notably indicating that NHL survivors who had more psychological problems also experienced lower HRQoL [39]. This suggests that comprehensive mood disorder management and improved guidelines for appropriate referral to psychological services could enhance HRQOL. Satisfaction with social support was associated with significantly greater scores on role emotional, mental health and social functioning. These findings suggest that more social support and supportive care are warranted during the follow-up of NHL survivors. [40] Socioeconomic deprivation was associated with poorer general health. Thus, to improve the HRQoL of NHL survivors, healthcare providers should consider social inequalities due to low financial capacity when planning for their care.

Clinical factors were not significantly associated with HRQoL except advanced Ann Arbor stages III and IV, which were associated with higher bodily pain. Similar results were found in another study that showed that BMI, type of lymphoma, systemic therapy and radiotherapy were not statistically significantly different for NHL survivors [41].

The strengths of our study are the use of validated instruments to assess HRQoL and psychosocial outcomes and the use of a specialized registry database, which had the advantage of being representative of regionally treated patients and avoiding potential selection bias. Furthermore, the response rate was high (63%). Moreover, to avoid non-response bias, we sent out reminders to increase the response rate. Lastly, independent variables were selected based on a priori knowledge of the risk factors for HRQoL and the backward elimination method with a *p*-value criterion of 0.157 was used to select the predictors to be included in the multivariable model. Additionally, complementary analysis was performed after multiple imputation and the results were almost identical to the main analysis, with lower R-square values than the analysis on complete data. However, this study had some limitations; notably, the cross-sectional design precluded documenting changes in HRQoL over time, and the study population was relatively small. In a later step, we will conduct a study including all registries specialized in hematological malignancies in France to consolidate the findings of our study. HRQoL two years later will also be investigated in Côte d’Or to assess changes in quality of life over time.

## 5. Conclusions

Our study found that, from 3 to 11 years post-diagnosis, the HRQoL of NHL survivors was poorer than in the French general population. Overall, NHL survivors had better mental HRQoL and poorer physical HRQoL. The main factors found to be associated with poor HRQoL of NHL survivors were age, sex, presence of anxiety, depression and economic problems. These findings suggest a need for supportive care to improve HRQoL, and consideration of these problems when developing care plans for NHL survivors.

## Figures and Tables

**Table 1:** Comparison of sociodemographic and clinical characteristics between respondents and non-respondents.

Characteristic	Respondents	Non-Respondents	*p*-Value
Age at time of survey, years			0.45
Mean (SD)	67 (12.4)	67 (18.3)	
Median {IQR}	69 {60–75}	71 56–80}	
Time since diagnosis, years			0.86
Mean (SD)	7 (2.3)	7 (2.2)	
Median {IQR}	6 {5–9}	6 {5–9}	
Sex N (%)			0.01
Women	71 (45)	56 (62)	
Men	86 (55)	35 (38)	
Histology type N (%)			0.06
FL	80 (51)	35 (38)	
DLBCL	77 (49)	56 (62)	
Ann Arbor stage N (%)			0.87
I–II	34 (22)	19 (21)	
III–IV	95 (61)	57 (63)	
Missing	28 (17)	15 (16)	
Treatment N (%)			0.74
R-CHOP chemotherapy	122 (77)	71 (78)	
Other	14 (9)	6 (7)	
No	20 (13)	14 (15)	
Missing	1 (1)	0	
Relapse post-treatment N (%)			0.07
Yes	11 (8)	2 (3)	
No	125 (92)	75 (97)	

*p*-value significant at <0.05; R-CHOP: Rituximab-Cyclophosphamide-Hydroxy Doxorubicin-Vincristine-Prednisone. IQR: interquartile range; SD: standard deviation.

**Table 2:** Sociodemographic, psychological and clinical characteristics of Non-Hodgkin Lymphoma Survivors.

Characteristic	Overall Population (N = 157)	Follicular Lymphoma (N = 80)	Diffuse Large B-Cell Lymphoma (N = 77)	*p*-Value
Age at time of survey in years				0.36
Mean (SD)	67 (12.4)	67 (10.1)	67 (14.4)	
Median {IQR}	69 {60–75}	68 {59–75}	70 {61–75}	
Time since diagnosis in years				0.35
Mean (SD)	6 (2.3)	6 (2.2)	6 (2.3)	
Median {IQR}	6 {4–8}	6 {4–8}	6 {5–8}	
Sex, N (%)				0.95
Women	71 (45)	36 (45)	35 (45)	
Men	86 (55)	44 (55)	42 (55)	
Marital status, N (%)				0.21
Married/living maritally	115 (73)	62 (78)	53 (69)	
Single	42 (27)	18 (22)	24 (31)	
Education level, N (%)				0.38
Primary or secondary	80 (51)	44 (55)	36 (46)	
University or higher	75 (48)	36 (45)	39 (51)	
Missing	2 (1)	0	2 (3)	
Lymphoma Ann Arbor stage, N (%)				0.90
I–II	34 (22)	15 (19)	19 (25)	
III–IV	95 (61)	43 (54)	52 (68)	
Missing	28 (17)	22 (27)	6 (7)	
Treatments, N (%)				<0.0001 *
R-CHOP chemotherapy	122 (77)	46 (57)	76 (99)	
Other ^†^	14 (9)	13 (16)	1 (1)	
None	20 (13)	20 (25)	0	
Missing	1 (1)	1 (1)	0	
Relapse post-treatment, N (%)				0.97
Yes	11 (8)	3 (5)	8 (10)	
No	125 (92)	56 (95)	69 (90)	
BMI at time of survey, N (%)				0.22
<18.5	5 (3)	1 (1)	4 (5)	
18.5–25	66 (42)	35 (44)	31 (40)	
25–30	50 (32)	24 (30)	26 (34)	
≥30	29 (19)	19 (24)	10 (13)	
Missing	7 (4)	1 (1)	6 (8)	
Comorbidity, N (%)				0.98
Yes	57 (36)	29 (36)	28 (36)	
No	100 (64)	51 (64)	49 (64)	
EPICES deprivation score ^§^, N (%)				0.20
<30	83 (53)	46 (57)	37 (48)	
≥30	50 (32)	22 (27)	28 (36)	
Missing	24 (15)	12 (16)	12 (16)	
Social support availability ^¶^, N (%)				0.46
<9	71 (45)	34 (41)	37 (48)	
≥9	78 (50)	42 (52)	36 (46)	
Missing	8 (5)	4 (5)	4 (6)	
Social support satisfaction ^¶^, N (%)				0.15
<30	41 (26)	17 (21)	24 (31)	
≥30	74 (47)	41 (51)	33 (43)	
Missing	42 (27)	22 (28)	20 (26)	
Anxiety ^¶¶^, N (%)				0.52
<11	129 (82)	68 (85)	61 (79)	
≥11	22 (14)	10 (13)	12 (16)	
Missing	6 (4)	2 (2)	4 (5)	
Depression ^¶¶^, N (%)				0.92
<11	136 (87)	70 (88)	66 (86)	
≥11	10 (6)	5 (6)	5 (6)	
Missing	11 (7)	5 (6)	6 (8)	
Sexual desire, N (%)				0.66
Increased	2 (1)	1 (1)	1 (1)	
Decreased/Lost	72 (46)	34 (43)	38 (49)	
Same	44 (28)	25 (31)	19 (25)	
Missing	39 (25)	20 (25)	19 (25)	

R-CHOP: Rituximab-Cyclophosphamide-Hydroxy Doxorubicin-Vincristine-Prednisone. IQR: interquartile range; SD: standard deviation. ^†^ Follicular lymphoma: radiotherapy (N = 4), surgery (N = 2), immunotherapy only (N = 7)/diffuse large B-cell lymphoma: chemotherapy only (N = 1). ^§^ Scores range from 0 to 100 and classify patients as deprived (≥30) or not deprived (<30). ^¶^ Availability scores range from 0 to 54 and satisfaction scores range from 6 to 36. A higher social support satisfaction score represents better perceived social support; social support availability and social support satisfaction were categorized according to their median value. ^¶¶^ The anxiety and depression subscores range from 0 to 21, with a score of 11 or higher indicating the probable presence of mood disorder. * Significant at *p*-value < 0.05.

**Table 3:** Characteristics of SF-12 subscale scores in Non-Hodgkin Lymphoma survivors.

SF-12 Scales	Overall Population		Follicular Lymphoma		Diffuse Large B-Cell Lymphoma	
	Mean (SD)	Median {x}	Mean (SD)	Median {x}	Mean (SD)	Median {x}
General health	56.4 (21.5)	60 {0–60}	57.8 (19.5)	60 {60–60}	54.9 (23.4)	60 {25–60}
Physical functioning	72 (35.1)	87.5 {50–100}	75.6 (33.2)	100 {50–100}	68.2 (36.8)	75 {50–100}
Role physical	60.2 (30.6)	62.50 {37.5–87.5}	65.3 (28.6)	62.5 {50–87.5}	54.8 (31.8)	56.2 {25–75}
Role emotional	63.9 (30.7)	75 {37.5–100}	68 (28.3)	75 {50–100}	59.5 (32.7)	62.5 {37.5–87.5}
Bodily pain	69 (30.1)	75 {50–100}	72.7 (29.6)	75 {50–100}	65.1 (30.3)	75 {50–100}
Mental health	64.4 (22.4)	62.50 {50–75}	67.6 (20.3)	75 {50–87.5}	61 (24)	62.5 {50–75}
Vitality	47.6 (25.5)	50 {25–75}	49.3 (26.8)	50 {25–75}	45.8 (24.3)	50 {25–50}
Social functioning	69.5 (28.3)	75 {50–100}	73.1 (26.6)	75 {50–100}	65.7 (30.7)	75 {50–100}
Mental Component Scale	44.6 (11.6)	46.2 {36.5–53.7}	45.6 (10.9)	46.3 {37.1–54.4}	43.5 (12.2)	44.1 {35.5–52.8}
Physical Component Scale	45.9 (10.1)	48.9 {40.2–52.6}	46.5 (9.9)	50.2 {40.2–54}	45.2 (10.5)	47.1 {37.8–52.3}

{x}: interquartile range (IQR).

**Table 4:** Multivariate linear regression model evaluating independent factors for the SF-12 subscale scores.

	GH		PF		RP		RE		BP		MH		VT		SF		MCS		PCS	
Independent Variable	β(SE)	*p* *	β(SE)	*p* *	β(SE)	*p* *	β(SE)	*p* *	β(SE)	*p* *	β(SE)	*p* *	β(SE)	*p* *	β(SE)	*p* *	β(SE)	*p* *	β(SE)	*p* *
Age at time of survey (years)			−1.1 (0.3)	<0.001													0.2 (0.09)	0.01	−0.3 (0.08)	0.002
Time since diagnosis (years)											2.7 (1.1)	0.01								
Sex														0.04						
Men													12.4 (6.1)							
Women													Ref							
Education level								0.01												
University or higher							14.1 (5.4)													
Primary or Secondary							Ref													
Relapse post-treatment														0.01						
Yes													−28.3 (11.1)							
No																				
Lymphoma Ann Arbor stage										0.006										
III–IV									25.3 (8.9)											
I–II									Ref											
Anxiety		0.02						0.01				<0.0001						0.001		
≥11	−14.3 (6.3)						−18.7 (7.5)				−27.1 (6.1)						−12.1 (3.4)			
<11	Ref						Ref				Ref						Ref			
Depression						<0.001		<0.001						0.002		0.02		0.001		
≥11					−37.2 (13.8)		−48.8 (11.2)						−36.6 (11.1)		−32.1 (13.8)		−16.3 (4.9)			
<11					Ref		Ref						Ref		Ref		Ref			
Social support satisfaction								<0.001				0.001				0.04		0.01		
≥30							23.8 (5.6)				17.3 (5.1)				15.7 (7.8)		7.1 (2.8)			
<30							Ref				Ref				Ref		Ref			
EPICES deprivation score		0.01								0.01										0.04
≥30	−12.8 (5.2)								−21.4 (8.5)										−5.1 (2.5)	
<30	Ref								Ref										Ref	

PF: physical functioning; RP: role limitations/physical health; RE role limitations/emotional problems; VT: vitality; MH: Mental Health; SF: social functioning; BP: bodily pain; GH: general health; MCS: Mental Component Scale; PCS: Physical Component Scale. Variables included in the multivariable model for each scale of SF-12: GH: age at time of survey, anxiety, EPICES deprivation score; PF: age at time of survey; RP: depression; RE: age at time of survey, time since diagnosis, education level, lymphoma Ann Arbor stage, anxiety, depression, BMI at time of survey, social support satisfaction; BP: time since diagnosis, marital status, lymphoma Ann Arbor stage, relapse post-treatment, EPICES deprivation score; MH: time since diagnosis, lymphoma Ann Arbor stage, anxiety, social support satisfaction; VT: sex, depression, relapse post-treatment, EPICES deprivation score; SF: anxiety, depression, social support satisfaction; MCS: age at time of survey, anxiety, depression, social support satisfaction; PCS: age at time of survey, lymphoma Ann Arbor stage, EPICES deprivation score. * *p*-value significant at <0.05; SE: Standard error.

## Data Availability

The dataset in this study is available from the first author upon reasonable request.

## Appendix A

**Table A1:** Multivariate Linear Regression Model Evaluating Independent Factors for the SF-12 Subscale Scores after multiple imputation.

	GH		PF		RP		RE		BP		MH		VT		SF		MCS		PCS	
Independent Variable	β(SE)	*p* *	β(SE)	*p* *	β(SE)	*p* *	β(SE)	*p* *	β(SE)	*p* *	β(SE)	*p* *	β(SE)	*p* *	β(SE)	*p* *	β(SE)	*p* *	β(SE)	*p* *
Age at time of survey (years)			−0.9 (0.2)	<0.001													0.2 (0.06)	0.003	−0.2 (0.07)	0.001
Time since diagnosis (years)											1.2 (0.8)	0.13								
Sex														0.09						
Men													2.2 (5.3)							
Women													Ref							
Education level								0.01												
University or higher							14.1 (5.4)													
Primary or Secondary							Ref													
Relapse post-treatment														0.12						
Yes													−8.5 (8.6)							
No																				
Lymphoma Ann Arbor stage										0.11										
III–IV									12.7 (8.1)											
I–II									Ref											
Anxiety		0.001						0.01				<0.0001						0.005		
≥11	−17.8 (4.7)						−18.7 (7.5)				−30.1 (5.5)						−9.1 (2.6)			
<11	Ref						Ref				Ref						Ref			
Depression						<0.001		<0.001						0.008		<0.001		<0.001		
≥11					−44.2 (9.1)		−48.8 (11.2)						−27.8 (10.5)		−35.7 (8.2)		−16.8 (3.2)			
<11					Ref		Ref						Ref		Ref		Ref			
Social support satisfaction								<0.001				0.002				0.003		<0.001		
≥30							23.9 (5.6)				12.2 (4.1)				16.6 (4.6)		10.3 (1.8)			
<30							Ref				Ref				Ref		Ref			
EPICES deprivation score		0.002								0.006										0.02
≥30	−10.7 (3.5)								−23.2 (8.4)										−4.4 (1.9)	
<30	Ref								Ref										Ref	

PF: physical functioning; RP: role limitations/physical health; RE role limitations/emotional problems; VT: vitality; MH: Mental Health; SF: social functioning; BP: bodily pain; GH: general health; MCS: Mental Component Scale; PCS: Physical Component Scale. Variables included in the multivariable model for each scale of SF-12: GH: age at time of survey, anxiety, EPICES deprivation score; PF: age at time of survey; RP: depression; RE: age at time of survey, time since diagnosis, education level, lymphoma Ann Arbor stage, anxiety, depression, BMI at time of survey, social support satisfaction; BP: time since diagnosis, marital status, lymphoma Ann Arbor stage, relapse post-treatment, EPICES deprivation score; MH: time since diagnosis, lymphoma Ann Arbor stage, anxiety, social support satisfaction; VT: sex, depression, relapse post-treatment, EPICES deprivation score; SF: anxiety, depression, social support satisfaction; MCS: age at time of survey, anxiety, depression, social support satisfaction; PCS: age at time of survey, lymphoma Ann Arbor stage, EPICES deprivation score. * *p*-value significant at <0.05; SE: Standard error.
